# Live Cell *in Vitro* and *in Vivo* Imaging Applications: Accelerating Drug Discovery

**DOI:** 10.3390/pharmaceutics3020141

**Published:** 2011-04-02

**Authors:** Beverley Isherwood, Paul Timpson, Ewan J McGhee, Kurt I Anderson, Marta Canel, Alan Serrels, Valerie G Brunton, Neil O Carragher

**Affiliations:** 1 Advanced Science & Technology Laboratory, AstraZeneca R&D Charnwood, Loughborough LE11 5RH, UK; E-Mail: beverley.isherwood@astrazeneca.com; 2 Beatson Institute for Cancer Research, Garscube Estate, Glasgow G61 1BD, UK; E-Mails: p.timpson@beatson.gla.ac.uk (P.T.); e.mcghee@beatson.gla.ac.uk (E.J.M.); k.anderson@beatson.gla.ac.uk (K.I.A.); 3 Edinburgh Cancer Research UK Centre, Institute of Genetics and Molecular Medicine, University of Edinburgh, Crewe Road South, Edinburgh, EH4 2XR, UK; E-Mails: m.canel@ed.ac.uk (M.C.); a.serrels@ed.ac.uk (A.S.); v.brunton@ed.ac.uk (V.G.B.)

**Keywords:** imaging, intravital, high-content, temporal, fluorescence, drug discovery, biomarkers, translation, efficacy

## Abstract

Dynamic regulation of specific molecular processes and cellular phenotypes in live cell systems reveal unique insights into cell fate and drug pharmacology that are not gained from traditional fixed endpoint assays. Recent advances in microscopic imaging platform technology combined with the development of novel optical biosensors and sophisticated image analysis solutions have increased the scope of live cell imaging applications in drug discovery. We highlight recent literature examples where live cell imaging has uncovered novel insight into biological mechanism or drug mode-of-action. We survey distinct types of optical biosensors and associated analytical methods for monitoring molecular dynamics, *in vitro* and *in vivo*. We describe the recent expansion of live cell imaging into automated target validation and drug screening activities through the development of dedicated brightfield and fluorescence kinetic imaging platforms. We provide specific examples of how temporal profiling of phenotypic response signatures using such kinetic imaging platforms can increase the value of *in vitro* high-content screening. Finally, we offer a prospective view of how further application and development of live cell imaging technology and reagents can accelerate preclinical lead optimization cycles and enhance the *in vitro* to *in vivo* translation of drug candidates.

## 1. Introduction: Challenges in Preclinical Drug Discovery

The past two decades have witnessed significant scientific and technical advances in the fields of drug discovery and translational medicine. Despite increased knowledge of disease mechanism and higher levels of financial investments in drug discovery research and development (R&D) the number of novel medicines approved by regulatory agencies such as the US Food and Drug Administration (FDA) has been in steady decline, with 50% fewer new molecular entities (NME) approved in the past five years compared with the previous five year period [1]. The development of truly innovative medicines that provide superior efficacy and safety in clinical settings over existing standards of care represents a formidable challenge. High attrition rates of candidate drugs in late stage preclinical development or clinical trials due to toxicity and limited efficacy suggests current pre-clinical drug discovery strategies are sub-optimal and poorly predict clinical outcome. The conventional target-directed drug discovery approach, where candidate drugs are developed on the basis of potency and selectivity against a single nominated target complements high-throughput serendipitous screening strategies of large chemical libraries. However, high costs, protracted timelines, low success rates and impending patent expirations suggest that the target directed drug discovery approach, on its own, no longer represents an effective R&D strategy.

Alternative drug discovery strategies that offer faster, more cost-effective and more opportunistic drug development approaches for new chemical entities, existing drug portfolios, multi-targeted agents and drug combinations are urgently required. A critical requirement of any new drug discovery approach is to provide greater predictivity of clinical response over existing methods and thus more productive translation of preclinical findings to clinically effective therapy. Recent advances in next generation sequencing, quantitative proteomics, RNAi technology, systems biology approaches and quantitative *in vitro* and *in vivo* imaging all embrace the biological complexity of disease and offer alternative strategies for target selection, target validation, candidate drug profiling and patient stratification [2,3]. Quantitative *in vitro* and *in vivo* imaging represents a more holistic approach to evaluation of drug efficacy, providing an unbiased and thus more opportunistic assessment of drug response in complex biological systems. Real-time image based analysis of drug response upon target activity and pathophysiology *in vitro* and *in vivo* may accelerate drug development timelines, reduce costs, provide novel mechanistic insight into adaptive response and increased clinical predictivity when applied appropriately to relevant model systems. In this review article we describe the latest research tools and approaches that facilitate live cell imaging and which are advancing the field of dynamic *in vitro* and *in vivo* imaging applications in drug discovery.

## 2. Live Cell Imaging *In Vitro*

Optical microscopes developed in the early 17th century represent the original transformative scientific technology for detailed physiological analysis of tissue and cell biology. Traditionally, microscopic imaging in research has been applied subjectively through manual application to small numbers of experimental or clinical specimens. However, recent developments of fully automated fluorescent microscopic acquisition platforms and associated image analysis algorithms have enhanced the throughput and statistical robustness of quantitative imaging. These advances have facilitated the integration of quantitative microscopic imaging into the drug development process and created the new discipline of high content screening. High content microscopic platforms and image analysis tools have evolved rapidly over the last 5 years and become established to different levels of sophistication and application within the pharmaceutical and biotechnology industry [4,5].

The widespread adoption of high content screening platforms by the pharmaceutical and biotechnology industry indicates a new trend in incorporating complex biological endpoints into drug discovery. High content screening is typically placed as a secondary screening strategy to confirm the quality of hits, identified through high throughput biochemical screening. However, high content assays are increasingly being applied to primary screening approaches, and have been proposed as a strategy to reduce attrition at later stages in drug development by frontloading the evaluation of novel target classes, chemical libraries and biologic therapeutics in more physiological cell systems [6]. Several publications describe the utility of high content imaging and analysis for profiling drug mechanism and defining structure activity relationship based upon phenotypic endpoints suggesting that quantitative imaging may offer an alternative approach to target-directed drug discovery [7-10]. The impact of high content biology on increasing the predictivity of the drug discovery process and transforming R&D productivity, in terms of delivering more successful therapies into the clinic, remains to be determined and will largely be dictated by the quality of the underlying biological models being subjected to investigation.

Typical high content cell-based screens employ fixed endpoint assays that provide a snapshot in time of a cellular response to pharmacological or molecular perturbation. In a similar manner to image-based immunohistochemical assessment of preclinical drug studies, such image based snapshots provide a reflection of pharmacological response at the time of sampling. Extension of image-based analysis to live cells, fresh tissue or live *in vivo* models in a time-resolved manner provides a more complete picture of dynamic biological processes through greater spatial and temporal understanding. Temporal analysis of drug response provides a number of unique advantages that enhance and complement fixed endpoint studies including: Quantification of transient phenotypic responses; Optimization of appropriate timepoints for endpoint studies; Interpretation of conflicting findings from endpoint studies that often arise from dynamic reversible processes that operate under precise temporal and spatial control; Characterization of adaptive responses; Determination of accurate scheduling and dosing regimes; Provision of additional data points of biological events collected over a time series, facilitating more robust quantitative analysis from less specimens. In the following sections of this article we shall review the development of the latest optical tools and enabling technology platforms, specifically optimized for live cell imaging applications *in vitro* and *in vivo*. We cite literature examples where live cell imaging has provided novel insight of pathophysiogical mechanism and drug response that cannot be extracted from fixed endpoint studies.

## 3. Live-cell Imaging *in Vitro*: Technical Advances and Novel Applications

### 3.1. Automated imaging platforms

Traditional time-lapse epifluorescent and confocal image acquisition microscopes provide detailed temporal and spatial analysis of cellular function. Throughput of such instruments is, however, restricted to one or a few samples per experiment, limiting their application within drug discovery. Most automated high content imaging microscopes can also be applied to live-cell kinetic studies where an environmental chamber is integrated onto the platform, however, throughput and analysis are once again rate-limited and environmental conditions are not optimal for long-term studies. Recent developments of more innovative microscopic configurations where the microscopic optical instrumentation is placed within a robust environmental chamber represent a significant technological advance in dynamic cell imaging. Such systems exemplified by IncuCyte™; Cell-IQ™ and Biostation CT™ incorporating software enabling remote control of, image acquisition, filter optic configurations and image analysis are optimized for long term kinetic studies across multiwell plates. This new generation of live-cell imaging microscopes increases the throughput and flexibility of examining dynamic cellular processes in response to multiple molecular or pharmacological intervention studies.

**Figure 1.**
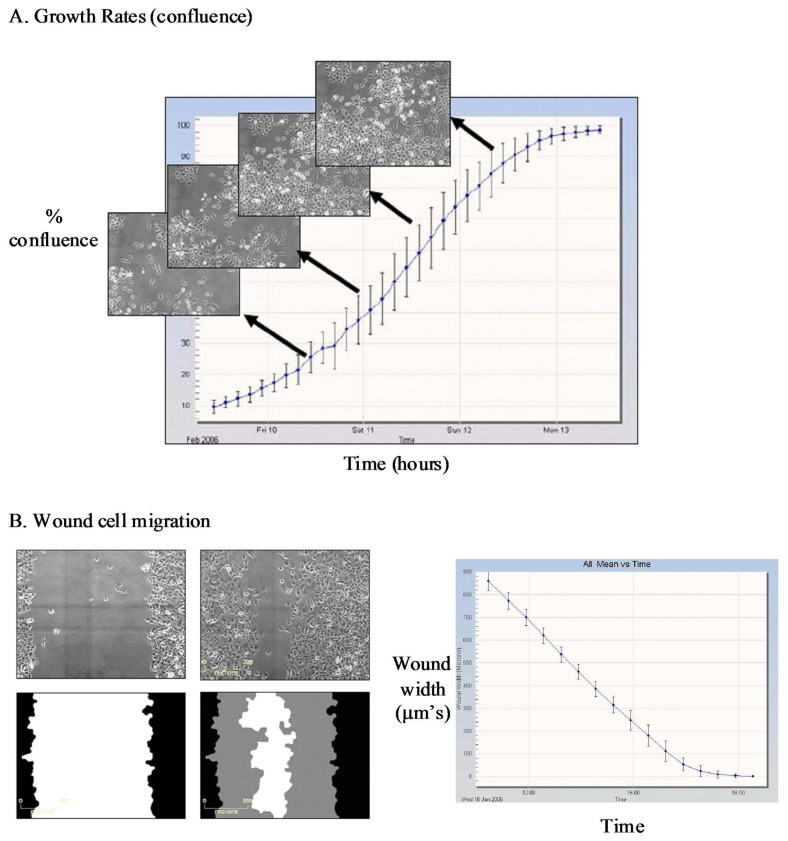

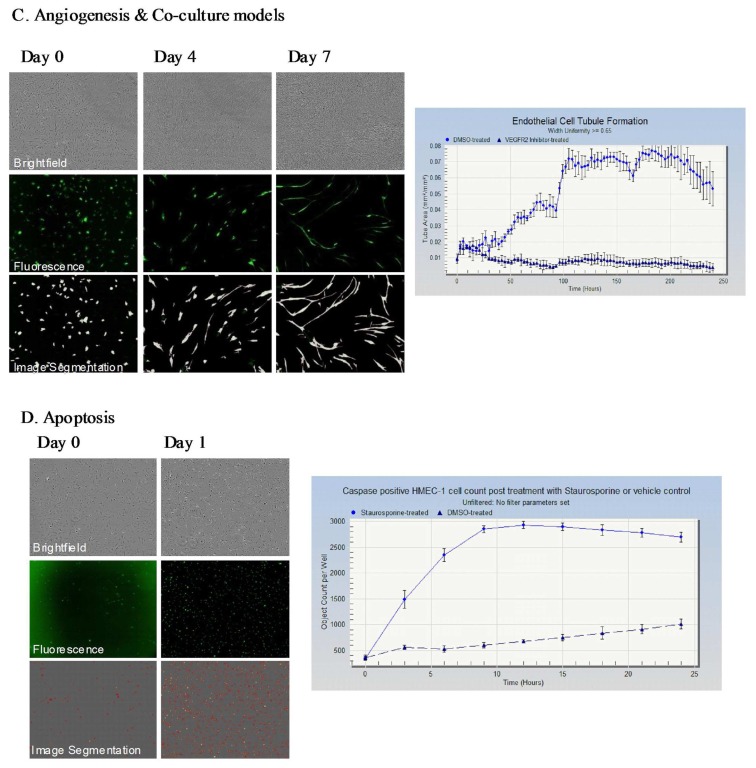
IncuCyte™ kinetic imaging platform and applications. Kinetic monitoring and quantification of (**A**) cell confluence, (**B**) cell migration following wounding of a monolayer of HT1080 fibrosarcoma cells. Data represents wound width (μm) at sequential time points quantified using the IncuCyte migration application module. (**C**) Angiogenesis in a human primary co-culture model of endothelial (HUVEC) and fibroblast cells where HUVECs have been labeled with GFP. Data represents cells treated in co-culture with VEGFR2 inhibitor (0.1 μM) or vehicle control for 10 days. Tubule area quantified using the IncuCyte angiogenesis algorithm. (**D**) Caspase-mediated apoptosis in a human micovascular endothelial cell line (HMEC-1) treated with Staurosporine (0.3 μM) and cultured in the presence of NucView™ caspase substrate. Caspase positive cells quantified using the IncuCyte object counting algorithm. n = 3 (error bars are standard deviation).

The IncuCyte-FLR™ is a compact, automated fluorescent and brightfield kinetic imaging platform that can be accommodated within a standard tissue culture incubator. The standard IncuCyte-FLR™ system can accommodate up to six 384-well plates to enable sequential monitoring of molecular and cell dynamics across multiple samples or drug treatment regimes in a single study. The IncuCyte™ platform also incorporates a suite of specific application modules comprising of image analysis algorithms and custom-designed consumables to facilitate automated monitoring of specific cellular processes such as cell growth, cell migration into a wounded monolayer, angiogenesis and apoptosis (Figure 1).

**Figure 2.**
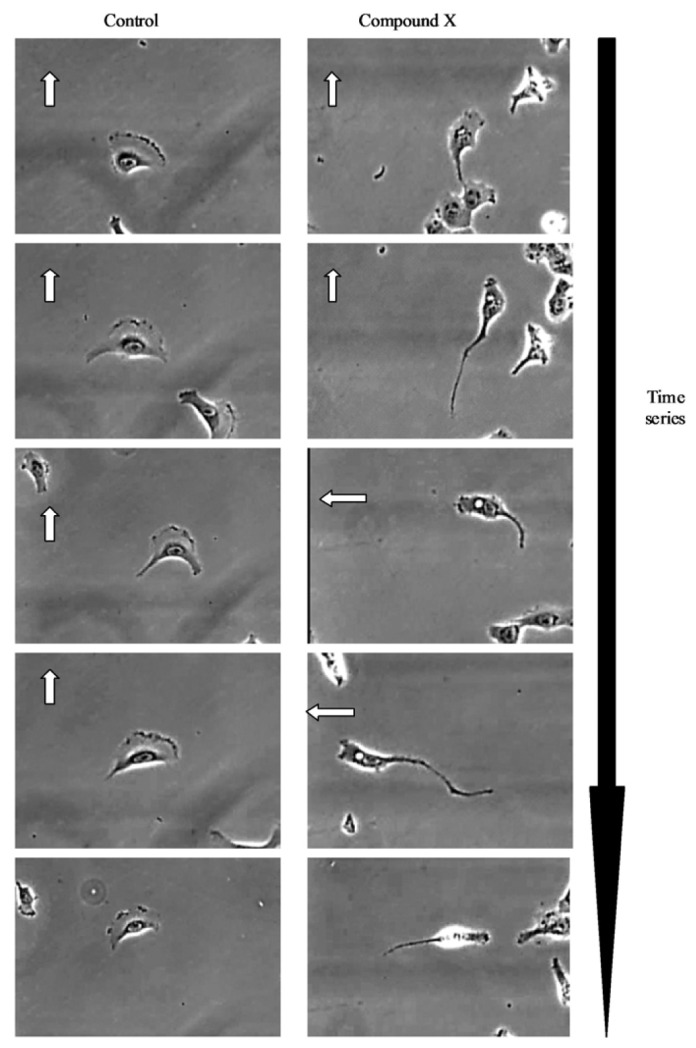
Kinetic profiling of cell migration response. Time lapse images obtained from the IncuCyte platform demonstrating the polarity and morphology of migrating HT1080 fibrosarcoma cells following compound treatment. White arrow denotes direction of cell movement. The time resolved images clearly demonstrate the impact that compound X has upon the adhesive mechanisms regulating cell motility with clear defects in lamellipodia formation at the leading edge and uropod retraction at the trailing edge (Figure 2).

Further visual inspection and detailed analysis of kinetic responses reveal additional information upon cellular physiology. For example, long-term monitoring of cell migration with the IncuCyte™, clearly distinguishes protrusion of the leading edge, lamellipodia dynamics and uropod (rear) retraction (Figure 2). Such analysis of dynamic cellular events provides insightful mechanistic information that can assist with defining drug or target mechanism-of-action studies.

**Figure 3.**
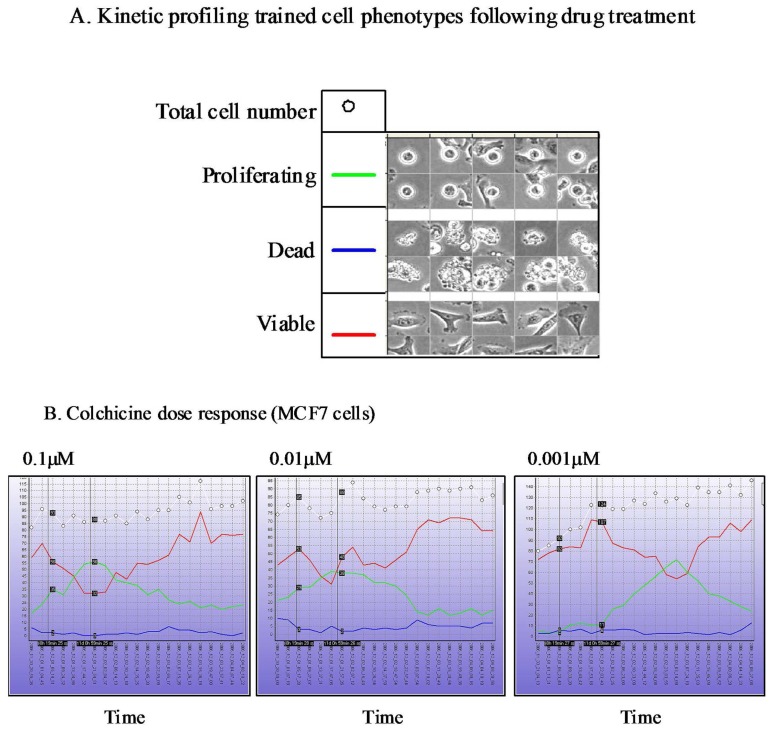
Cell-IQ™ Kinetic phenotypic profiling of drug response. (**A**) Machine learning based training of cellular phenotypes from phase contrast images. (**B**) Kinetic monitoring of the proportion of each phenotypic class and total cell number (within a single field of view) following treatment with different concentrations of colchicine. Data presented provides quantitative analysis of transient mitotic arrest phenotype.

The Cell-IQ™ represents a fully integrated incubator, phase contrast and fluorescent image acquisition system linked to image analysis software based upon “machine vision” artificial intelligence approaches. The Cell-IQ™ acquisition software provides a flexible interface enabling fully automated or user defined image acquisition across 2D and 3D cell culture systems. The use of machine vision based training provides a flexible approach to monitoring proportions of distinct cellular phenotypes within complex samples such as co-culture models. In addition to classifying phenotypes other processes such as cell attachment, migration velocity, migration direction, neurite outgrowth, vesicle formation, angiogenesis and stem cell differentiation have all been recorded on the Cell-IQ™ platform. The Cell-IQ™ system enables robust kinetic profiling of phenotypic response following drug treatment including transient and long-term events (Figure 3). The temporal profile of phenotypic response and transient events often vary between cell type, drugs and drug doses (Figure 3). Thus, kinetic profiling provides robust phenotypic analysis of drug response and facilitates the selection of the most appropriate timepoints for standard high-content endpoint screens.

The Nikon Biostation CT™ platform represents the first multi-objective fluorescent and phase contrast microscope integrated with automated plate handling robotics all within a cell-culture incubator. Such advances in kinetic imaging platforms have the potential to significantly increase the throughput and scope of live-cell studies by enabling more extensive and robust target identification, validation and secondary screening applications.

The examples provided here highlight the capabilities for new kinetic imaging technology to accurately record adapting transient responses that may also include dynamic flux through signaling pathways or temporal/spatial oscillations that are not readily detected by standard endpoints. The additional information provided by such temporal analysis is likely to provide further insight into drug or target mode-of-action and cross-talk between distinct pathways and mechanisms. The temporal analysis of phenotypic response also provides valuable insight into pharmacodynamic properties of candidate drugs. By relating the kinetics of pharmacodynamic phenotypic response to known *in vivo* pharmacokinetic properties of drug candidates shall facilitate more optimal scheduling and dosing strategies for *in vivo* studies. Thus, such technologies may drive the development of a new class of kinetic biomarkers that can be placed earlier in the drug discovery process to ensure more successful translation towards *in vivo* efficacy.

### 3.2. Multidimensional imaging

Multiphoton confocal microscopy offers a number of advantages over conventional fluorescent microscopy methods for live cell imaging such as greater depth penetration, better Z-resolution improving 3D reconstruction, less phototoxicity and photobleaching of samples. These advances facilitate the long term analysis of cell behavior in live-cell cultures and provide more sensitive and quantitative analysis of fluorescent reporters or labeled cells within more physiological 3D culture systems, thick tissue samples or live *in vivo* models. Cell migration assays traditionally used for monitoring the effect of drug treatments upon cell motility include Boyden chamber type transmigration or scratch wound endpoint assays [11,12]. While such assays provide a robust quantification of the number and relative proportions of migrating cells they provide limited mechanistic information on cell motility or drug response. Live cell imaging studies have provided additional mechanistic insight into heterogeneous mechanisms of cell motility and the effect of drug response upon such mechanisms in both 2D and 3D culture systems [13,14]. Multiphoton confocal analysis has further characterized several distinct modes of tumor invasion that occur in more physiological 3D environments and categorized these as, mesenchymal, amoeboid and collective [15-17]. Mesenchymal tumor invasion is characterized by single cell locomotion associated with integrin mediated adhesion and matrix metalloproteinase (MMP)–mediated remodeling of extracellular matrix (ECM) [15]. Amoeboid invasion is characterized by a spherical, single cell, locomotion phenotype that is independent of MMP activity [18]. Collective invasion is characterized by tumor cell locomotion as a multicellular sheet associated with both integrin mediated adhesion and MMP-mediated ECM remodeling. These dynamic imaging studies shed new light on the morphological and functional characteristics of multiple tumor invasion mechanisms. Plasticity between distinct tumor invasion mechanisms enables rapid adaption of tumor invasion in response to environmental factors including pharmacological intervention. Such mechanistic adaptation may explain conflicting pharmacological responses observed between distinct tumor cell models and recorded clinical resistance to potential anti-invasive therapies such as MMP inhibitors [19]. Further studies using live cell confocal microscopy uncover the influence that stromal cell types, within the tumor microenvironment, have upon tumor invasion [20]. MMP dependent invasion of fibroblasts through 3D ECM preparations creates tracks in the ECM environment that facilitates the collective invasion of squamous cell carcinoma cells through such tracks [20]. These live-cell imaging studies, therefore, uncover a protumorigenic role for stromal cell remodeling of ECM topology to create a more permissive microenvironment for tumor invasion. Through further discovery, phenotypic characterization and quantification of distinct tumor invasion mechanisms by live-cell imaging, drug discovery researchers can begin to tailor their experimental models appropriately to specific target classes or to recapitulate multiple drug resistance mechanisms to aid the evaluation of new pharmacological interventions that counteract such adaptive responses.

## 4. Advanced Imaging Technologies

### 4.1. Photactivation, photoswitching, photobleaching and photoinactivation

Serial site-directed amino acid mutation studies have created genetically engineered fluorescent proteins that are photoswitchable from dark to bright, or from one color to another following excitation at a specific wavelength [21]. Such photoactivatable probes can be used in live cell systems to track cell movement, monitor protein diffusion dynamics, protein stability and cell-substructure function [22-28]. Fluorescence recovery after photobleaching (FRAP) offers an alternative approach for monitoring protein diffusion and dynamic intracellular processes. FRAP analysis of GFP labeled integrin subunits at sites of cell-substrate adhesion has been applied as a quantitative measurement of the dynamic turnover of integrin associated adhesions, thus providing both spatial and temporal analysis of cell adhesion mechanisms not provided by traditional adhesion assays or endpoint measurements [29].

Photo-reactive proteins can also be exploited to positively manipulate protein activity. Recently, the photo-reactive domain of phototropin was fused to a constitutively active mutant of Rac1, causing sterical hindrance and impairing its interaction with downstream effectors [30]. Upon light illumination, the photo-reactive domain unwinds and allows the un-caging of the fused protein, leading to Rac1 activation. Using this approach light-guided timing and location of protein activation can be precisely regulated at sub-cellular resolution. This method has recently been used to show light-mediated guidance and control of cells movement both *in vitro* and *in vivo* [30,31]. Similarly, adaptations of light-gated proteins to control upstream regulators of the Rho family GTPases have recently been documented to control cell protrusion and migration [32]. Chromophore assisted light inactivation (CALI) is a specific technique for locally perturbing protein function through the generation of reactive oxygen species (ROS) that destroy specific proteins following excitation of an attached fluorophore. This approach provides a precise mechanism for inactivating protein targets with improved spatial and temporal control than provided by popular methods of genetic knockout or siRNA [33]. Using GFP as a CALI sensor, optical irradiation of GFP-α-actinin but not GFP-FAK (focal adhesion kinase) resulted in a loss of actin-fiber association with integrins. These are the first studies to demonstrate that FAK is not critical for stress fiber attachment to integrin mediated cellular adhesions [33]. The application of light-mediated activation/inactivation of other proteins both spatially and temporally in complex biological systems will provide a diverse platform in which to validate targets and investigate various drug regimes under appropriate biological context.

### 4.2. FRET and FLIM

Forster Resonance Energy Transfer (FRET) is a technique that monitors the transfer of energy from a donor to an acceptor fluorophore when donor-acceptor fluorescent pairs come into close proximity. FRET is performed following specific excitation of the donor flurorophore, and subsequent analysis of the fluorescent emission properties of the acceptor fluorphore. Alternatively, a change in the fluorescent decay rate of the donor can be used to measure FRET and changes in the fluorescent lifetime of the donor can be recorded by Fluorescent Lifetime Imaging Microscopy (FLIM) methods. The exponential decay rate of the fluorophore signal, rather than its intensity, is used to create the images in FLIM and can provide a more robust FRET readout due to less scattering of light or other artifacts associated with acceptor excitation/emission. Each fluorophore has unique decay rates, thus FLIM can also be used to distinguish between fluorophores in a multiplex assay. The exponential decay of fluorophores or autofluorescent biological structures can be altered by physiological events and thus label or label-free FLIM may provide an indirect readout of physiological modifications.

Both FRET and FLIM have been applied to monitor, protein-protein interactions, conformational changes in tertiary protein structure or proteolytic cleavage events in live cells [34]. Unlike ratiometric FRET, FLIM is independent of the donor concentration and therefore insensitive to bleaching. Specific FRET biosensors have been designed to monitor, in live cells, the activity of a number of distinct kinase (Src, FAK, EGFR), protease targets (calpain) [35-37] and intracellular signaling of calcium [38,39] amongst others [40]. The most common method used for quantification of FLIM reporters is Time Correlated Single Photon Counting (TCSPC). However, the application of longitudinal TCSPC FLIM to live cell applications is limited by long acquisition times particularly across large fields of view or multiple Z-planes. Thus, multiple acquisition of TCSPC FLIM in live cell systems is associated with a high risk of photobleaching and photodamage. A new innovation in FLIM technology is the development of, wide-field, time-gated, imaging using a gated optical intensifier (GOI) combined with a Nipkow disc confocal microscope that significantly reduces the time for FLIM data acquisition to 10 frames per second [41]. These advances have led to the development of high-speed FLIM systems suitable for high-content analysis of FLIM reporters in live cell and 3D model systems across multiple samples [42]. High-speed FLIM has been applied to monitor the interaction of Ras protein with Raf binding partners, a key event in the RAS-MAPK signaling cascade, in live-cell specimens in a format suitable for chemical or molecular screening [42]. Furthermore, such approaches have permitted a multiplexed FRET readout of the spatial and temporal resolution of both Ras activation and calcium flux in a single live cell assay [43]. Similarly, the simultaneous investigation of intensity based FRET probes within the same cells has recently been used to dissect the synchronized activity of individual RhoGTPases during coordinated cell migration [44]. Frequency domain based-FLIM has also been adapted to allow for fast FLIM in a 96-well high-throughput format for the *in situ* identification of tyrosine phosphorylation networks downstream of EGFR signaling [45]. The potential adaptation of these techniques for the simultaneous analysis of different probes within the same cell in a high throughput setting will enable the precise temporal and spatial monitoring of target activity and associated downstream or compensatory signaling pathways in response to a variety of drug screening regimes.

**Figure 4.**
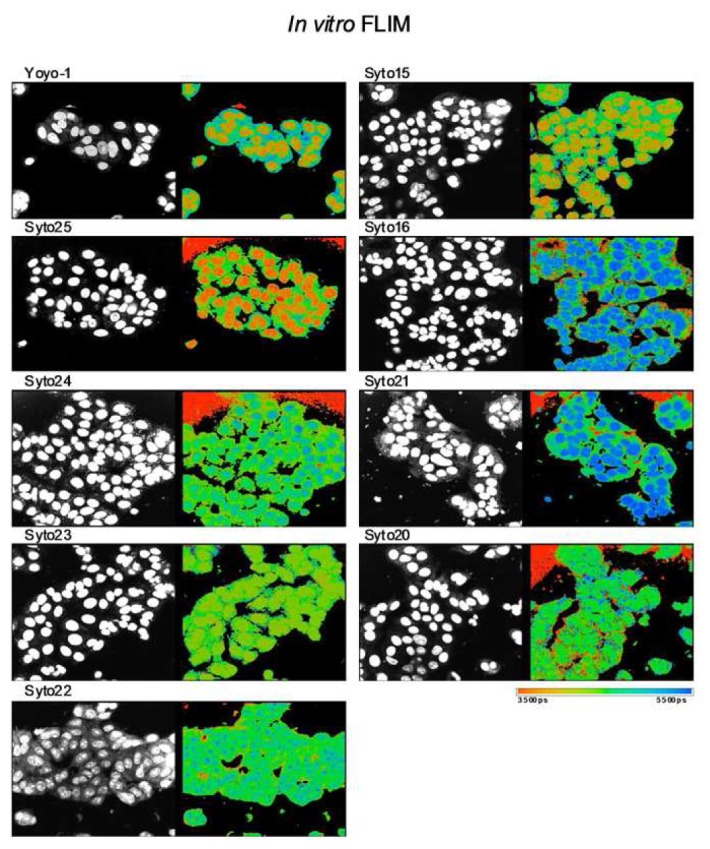
*In vitro* Fluorescent Lifetime Imaging Microscopy (FLIM): Discrimination of cellular DNA and RNA. Intensity (left panel) and lifetime images (right panel) of MCF-7 cells stained with a range of green-fluorescent nucleic acid binding dyes. The dyes are commonly used as nuclear or cell counter stains in standard fluorescent microscopy (left panel) but are known to differ from each other in one or more characteristics, including DNA/RNA binding selectivity. Here, additional contrast provided by FLIM of Syto15 and Yoyo-1 distinguished cytoplasm, nucleus and nucleoli. Contrast observed with Yoyo-1 is due to longer lifetime of dye bound to RNA and shorter lifetime of dye bound to DNA (data not shown). FLIM contrast observed with other dyes was either minimal (Syto23) or offered less contrast across cytoplasmic, nuclear and nucleolar compartments (Syto25, Syto16, Syto21, Syto22, Syto20). This study identified dyes appropriate for rapid cell image segmentation using FLIM rather than requiring dye multiplexing in either live or fixed cells and suggests that additional screening to identify FLIM contrast for biological event monitoring e.g. apoptosis or cellular differentiation may be worthwhile. Images acquired by Clifford Talbot *et al.* in the Photonics Group at Imperial College London using a laser scanning confocal TCSPC microscope.

Lifetime imaging has recently been employed to compare the intrinsic lifetime of ECM proteins within spatially distinct regions of human tissue, such as the core or the periphery of a tumor [46]. Furthermore, this technique has been applied to evaluate invasive and non-invasive stromal tissue in fixed or freshly excised human cancer samples [47]. Lifetime imaging in this manner can provide vital intrinsic, contextual, and local environmental information on levels of hypoxia, NADH, the constitution of ECM components, and the overall metabolic status at specific areas within the tumor, such as detecting necrotic region within the tumor. This application of lifetime imaging to provide a histological profile which can document changes in the local tissue micro-environment upon therapeutic intervention will be of use in the assessment of drug delivery. Recently, lifetime imaging has been utilized in human tissue microarray (TMA) studies to understand not only the tumor status but the environment in which different tumor types prosper [48]. FLIM has also been used to obtain additional contrast from cells labeled with nuclear dyes (Figure 4) and properties of such dyes have been used to detect apoptosis [49] and identify changes in DNA/RNA content for the study of cellular proliferation, differentiation and cell cycle [50,51]. Lipid-targeting fluorescent probes (e.g. di-4-ANEPPDHQ) have also been used for FLIM applications in live cells to probe lipid order in the cellular membrane [42,52].

### 4.3. Fluorescent protein complementation

An alternative approach for monitoring protein-protein interactions dynamically in live cells is through the application of bimolecular fluorescent complementation (BiFC) assays [53]. BiFC monitors protein-protein interactions by expressing a split fragment of a fluorescent reporter protein on each individual protein binding partner. When the protein partners interact the split fluorescent reporter proteins complement together forming a functional fluorescent molecule that acts as a positive reporter for the protein-protein interaction. Engineered panels of cell lines expressing multiple fluorescent protein complementation pairs enables the dynamic profiling of multiple pathways in live cells [54].

The methods described above exploit the unique spectral properties of fluorescent molecules combined with the spatial and temporal resolution provided by microscopic imaging to enable detailed kinetic and spatial analysis of pathway events and cell physiology in live cell systems. These approaches provide additional insight and quantitative analysis of dynamic transient events that are not obtained from fixed endpoint studies. Specific applications of live cell imaging demonstrating added value over fixed endpoint studies include, cell migration, cell-cycle progression, endocytosis, autophagy, subcellular translocation, cell-cell interactions, intracellular calcium fluxes, oscillations in biochemical signaling and turnover rates of cell adhesions [55-58]. Extension of such live-cell analysis approaches to *in vitro* and *in vivo* preclinical drug discovery shall enable a more comprehensive temporal evaluation of drug response in complex biological systems. Integration of fluorescent reporter molecules and spectroscopic techniques with dedicated *in vitro* and *in vivo* live optical imaging systems shall further enhance the application of live-cell imaging into preclinical drug discovery.

## 5. Live Cell Imaging *In Vivo*

Complimentary advances in advanced imaging technologies, near-infrared fluorescent NIRF imaging reagents, intravital microscopic methods and dedicated *in vivo* optical imaging systems have driven the application of fluorescent imaging into live animal models and fresh tissue specimens [59-61]. The development and commercial availability of a variety of near-infrared fluorophores and nanoparticles optimized for *in vivo* application (included in Table 1) has stimulated the development and adoption of optical *in vivo* imaging for preclinical drug discovery. Longitudinal *in vivo* imaging studies in live animals provide more detailed information from fewer animals at significant cost savings compared with traditional preclinical methods thus, representing an attractive approach for preclinical drug evaluation studies.

### 5.1. Non invasive in vivo imaging

**Figure 5.**
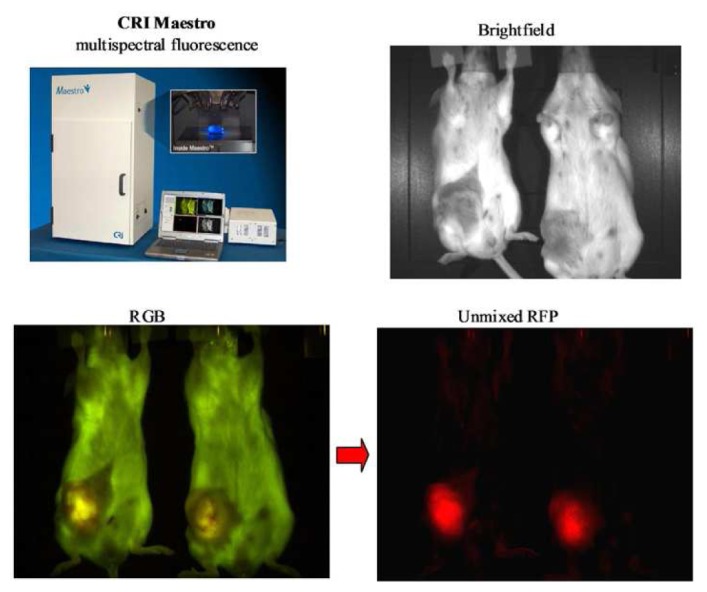
Multispectral *in vivo* imaging. Application of the Maestro™ system to detect and differentiate RFP labeled MDA-MB231 breast cancer cells in a live mouse following orthotopic implantation in the mammary fat pad.

Recent advances in *in vivo* fluorescent imaging technologies include; multispectral imaging, tomographic reconstruction and high resolution intravital multiphoton confocal microscopy. Specific examples of dedicated fluorescent *in vivo* imaging systems offering non-invasive analysis of fluorescent reporters include the Maestro™ system from Cambridge Research & Instrumentation, Inc. (CRi). The Maestro™ platform incorporates a liquid crystal tunable filter and multispectral unmixing software to facilitate subtraction of background autofluorescence and multiplexing of multiple overlapping fluorophores *in vivo*. The increase in signal to noise associated with spectral unmixing can increase the sensitivity of analyzing distinct fluorescent signals *in vivo* when compared with standard monochrome reflectance imaging approaches [62] (Figure 5). The recent development of the Maestro dynamic™ system further facilitates the acquisition of temporal multispectral data enabling quantitative assessment of the biodistribution and pharmacokinetic properties of multiple probes in a sequential manner. Advances in the development of more sensitive charge coupled device (CCD) cameras linked to optimized photon amplification and counting technology such as that exemplified by the Photon Imager from Biospace Lab offer maximal sensitivity for both bioluminescent and fluorescent *in vivo* imaging. The intensified CCD technology within the Photon Imager amplifies all light emitted from the specimen by a sequential process of converting photons to electrons, electron amplification and conversion back to photons by a phosphorescent screen. As a direct result of this process the CCD camera in the Photon Imager is able to record and forward the optical signal emitted from *in vivo* samples at a rapid constant frame rate of 43 Hz facilitating real-time analysis of optical reporters. A key consideration for the detection and quantification of optical probes *in vivo* or in thick tissue samples is the scattering of light by biological tissue limiting quantitative assessment of signal strength, size and depth. Advances in fluorescent molecular tomography systems such as the FMT2500 LX, commercialized initially by VisEn Medical and subsequently by Perkin Elmer, provides a transmitted light excitation and emission configuration and associated software that compensates for the scattering of light through tissue. The FMT reconstruction software provides a more accurate assessment of the light emitting signal including robust quantification of size and depth of fluorescent reporters at any position in a mouse model [63]. The Ivis® spectrum and Ivis® Kinetic instruments from Caliper represent fully integrated non-invasive optical devices that facilitate both non invasive bioluminescent and fluorescent imaging in small animal models. The Ivis® systems are highly versatile platforms offering both spectral unmixing and single view 3D tomographic capabilities. The added sensitivity provided by a highly sensitive electron multiplying charge coupled device (EMCCD) camera on the Ivis®Kinetic system enables acquisition of bioluminescent or fluorescent reporter signals in milliseconds facilitating real-time analysis of biologically relevant events recorded by optical biosensors. The development and application of non-invasive *in vivo* optical imaging systems described above facilitate the longitudinal study of physiological responses, pharmacokinetics and pharmacodynamics in preclinical models without the need for sacrificing the animal. However, the application of non-invasive imaging is limited by the spatial resolution of images obtained and the availability of optimized probe sets.

### 5.2. High resolution intravital imaging

Advances in intravital confocal microscopy that provide cellular and subcellular resolution of biological events *in vivo*, more akin to *in vitro* high-content studies, provide a more adaptable platform for the development of live-cell *in vivo* imaging applications [61]. A number of recent technical developments have facilitated high resolution confocal microscopy in live *in vivo* models. The design of new lens configurations such as the microprobe lenses supplied by Olympus and miniaturized fiber optic fluorescent microscopic endoscopic (FME) devices facilitate physical access and functional analysis of deep tissue sites [64]. Such technology has been further developed to enable multidimensional fluorescent imaging *in vivo* with the introduction of fluorescent lifetime measurement capability on the Cellvizio endomicroscope platform [65].

**Figure 6.**
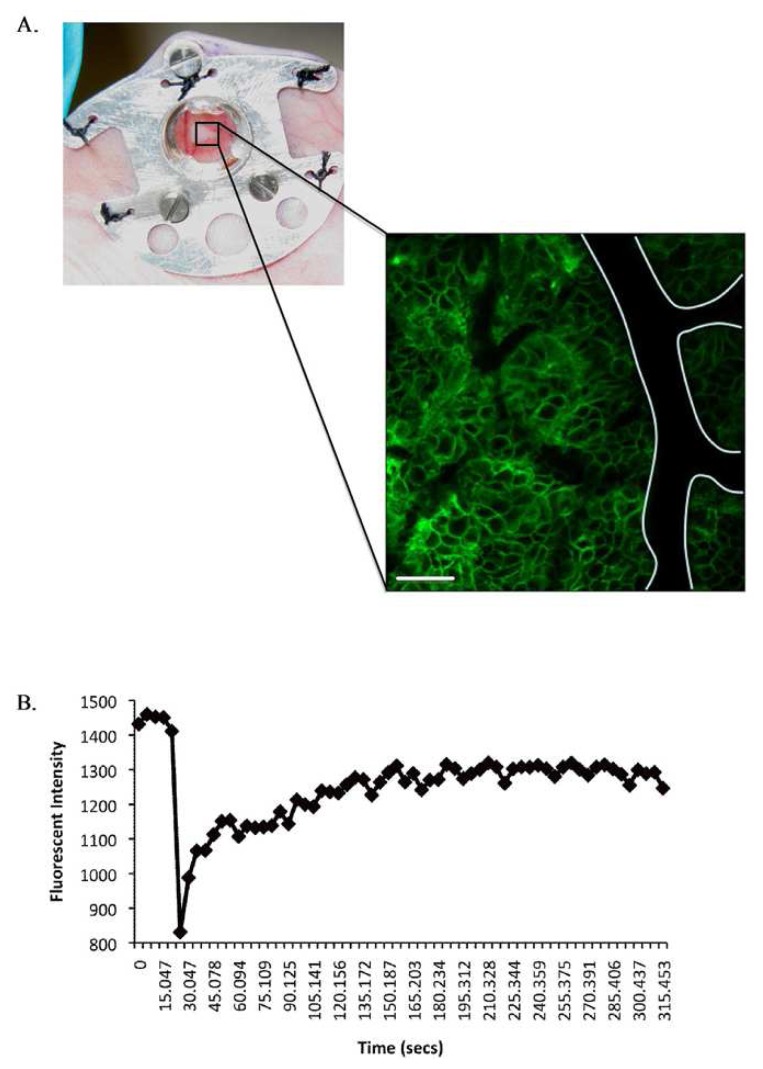
Intravital *in vivo* imaging and Fluorescence recovery after photobleaching (FRAP) to monitor E-cadherin dynamics. (**A**) Example of an optical window chamber containing a tumor implanted on the back of a CD-1 nude mouse. Inset: confocal image of GFP-E-cadherin expressing tumor cells acquired using optical window chambers, a major blood vessel is outlined. Scale bar 50 μm. (**B**) A representative FRAP recovery curve after photobleaching of a region of GFP-E-cadherin at the plasma membrane *in vivo*.

Implantation of tissue window devices such as optically clear glass coverslips in the skin enable long-term repeated high-resolution *in vivo* imaging without the elicitation of artifactual inflammatory responses associated with surgical trauma at time of image acquisition (Figure 6A). The design and application of tissue window devices for live *in vivo* imaging represents an increasing trend as demonstrated by a number of recent publications. Several studies describe the design of tissue window devices tailored to specific tissue sites under examination, including cranial windows [66], mammary windows [23] and dorsal skinfold windows [26,67]. Tissue window devices offer a number of advantages over alternative surgical exposure methods including the so called “skin flap” method including, sample stability, maintenance of tumor environment and repeated long term imaging in conjunction with recoverable anesthesia. Furthermore, in our experience the mechanical stability offered by the use of dorsal skinfold windows is of significant benefit when acquiring photobleaching/photoactivation time series. Two important considerations in the application of tissue window devices are the surgical methods of implantation to ensure minimal tissue damage and proper sterilization prior to use including anti-bacterial and anti-fungal treatment. Further optimization of the design and commercial availability of more cost effective and improved tissue window devices in association with approved surgical implantation protocols will provide a significant stimulus to the field of “live” *in vivo* imaging. Specific advantages of applying multiphoton confocal approaches together with intravital microscopy include, enhanced spatial resolution, and reduced image degradation due to light scattering. Greater depth penetration through tissue, improved optical efficiency, better z-registration for 3D reconstruction and less photodamage and phototoxicity enabling longer acquisition time and repeated imaging in live specimens. Multiphoton imaging can also provide additional detail regarding interactions with the surrounding extracellular tissue microenvironment provided by second harmonic signal generation which highlights non-centrosymmetric protein structures such as collagen and elastin.

Specialist microscopy systems that are optimized to facilitate the high resolution imaging of live animals are now reaching maturity. One example is the multiphoton multi-point scanning TriM-scope from LaVision Biotec. The design rational behind the system is to enhance the acquisition of optically sectioned imaging of thick samples at depths exceeding those possible via standard confocal microscopes. The TriM-scope achieves this by enabling customization of multiple imaging configurations tailored to the sample and experiment goals. This flexibility enables the scanner unit to couple into either an upright or inverted microscope body, accept the free space coupling of multiple excitation sources and multi-point beam scanning and detector types and locations optimized to the specimen being imaged.

The TriM-scope has two light input ports and the type of sources used are typically in the pulsed titanium sapphire (Ti:Sapphire) or optical parametric oscillators (OPO) class of laser. OPO cavities are pumped by the IR output from a Ti:Sapphire laser and which, through the choice of optical crystal, can produce excitation source light up to 1,400 nm. The generation of far-red shifted excitation has the obvious advantage of reduced scattering and absorption while passing through dense tissue, but also enables excitation of fluorescent probes in the NIR resulting in fluorescence that also suffers less scattering and absorption than the typically used GFPs. In addition to optimizing the delivery of excitation light, the TriM-scope design optimizes the detection of generated light through the placement of sensitive photo-multiplier tube (PMT) detectors as close to the sample as possible. By placing the PMT detectors directly behind the objective in both epi- or trans-illumination geometry the optical emission path length is significantly reduced when compared with standard confocal microscopes, thereby, maximizing sensitivity. However, if the experimental priority is the rate of acquisition, for example kinetic monitoring of drug responses, instead of depth penetration or sensitivity, then the option of multiplexing the excitation beam into a maximum of 64 separate beams allows the generation of multiple emission spots which can be imaged simultaneously using a CCD camera. Beam-splitting reduces the time to image a region of interest compared to imaging with a single beam, however, this approach is associated with a reduction in the power per excitation spot as it is distributed between the generated spot pattern, meaning that scattering will have a more apparent effect.

Research in several disease areas have benefited significantly from intravital confocal microscopy of preclinical models, most notably tumor biology. Pioneering intravital microscopy studies on tumor invasion *in vivo* have provided novel mechanistic insights with new pro-invasive roles being assigned to inflammatory macrophages and collagen fibres [68-70]. Recent studies exploiting the unique photoactivation and photobleaching properties of fluorescent probes have provided further mechanistic insight into specific biological mechanisms of disease and therapeutic response [26,27]. The application of optical windows in conjunction with localized photobleaching of GFP-labeled E-cadherin expressed at adherens junctions in engineered tumor cells has been used to monitor E-cadherin dynamics both *in vitro* and *in vivo* [27] (Figure 6). E-cadherin represents a family of transmembrane cell adhesion molecules that bind to the E-cadherin molecules on adjacent cells to mediate cell-cell adhesion. Thus, dynamics play a critical role in regulating inter-cellular adhesion between epithelial cells and many epithelial derived tumors. Experimental validation demonstrates that quantification of E-cadherin mobility into photobleached areas provides a preclinical measure of E-cadherin function and cell adhesion strength. E-cadherin mobility recorded, following exponential curve fitting, as the half-time of recovery provides a rapid and robust quantitative measure of E-cadherin function and inter-cellular adhesion *in vivo* [27] (Figure 6B). Photoactivation of a caged (non-fluorescent) plasma membrane targeting probe provides quantitative assessment of protein diffusion at the plasma membrane and in parallel with photobleaching can be used to quantify the proportion of the immobile fraction of protein stabilized at cell-cell junctions [27]. These approaches have been used to confirm the mechanism-of-action of small molecule kinase inhibitors [26,71].

The instantaneous readouts provided by such intravital imaging methods are in stark contrast to long-term metastasis models that can take up to several months to assess tumor invasion. Rapid image based evaluation of drug responses directly within the tumors of live animals facilitate more accurate scheduling and dosing, require fewer animals and accelerates the evaluation of preclinical response enabling the chemical optimization of lead compounds based on quantifiable *in vivo* biological endpoints. Interestingly, the dynamics of E-cadherin-GFP and plasma membrane diffusion rates in tumor cells implanted *in vivo* differ significantly between identical cell clones cultured on *in vitro* 2D culture substrates [27]. In addition, the observed effect of a licensed kinase inhibitor drug, dasatinib upon increasing the immobile fraction of E-cadherin *in vivo* was not observed when the cells where cultured on standard 2D plastic substrates. Critically, these studies demonstrate that 2D *in vitro* culture conditions do not accurately recapitulate the *in vivo* pharmacological mechanism-of-action of dasatinib and possibly other drugs, suggesting that 2D *in vitro* cell based models commonly used in high-content biology poorly predict *in vivo* drug response. The application of precise image-based monitoring of protein dynamics and physiological responses *in vivo* and *in vitro* may help calibrate the predictivity of *in vitro* model systems and ensure development of more predictive *in vitro* high content assays.

**Figure 7.**
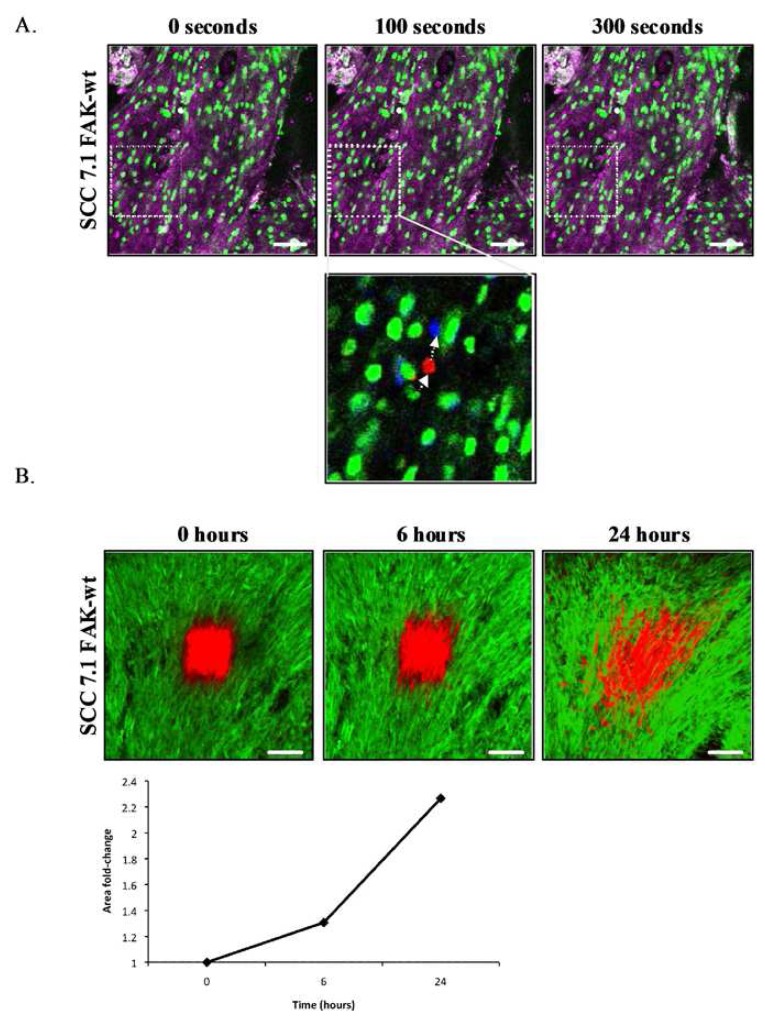
Intravital *in vivo* photoswitching to monitor tumor invasion. (**A**) Images from a time series acquired from a GFP expressing squamous cell carcinoma (SCC) xenograft expressing wild-type FAK (green: GFP, purple: confocal reflectance of extracellular tissue), scale bar 50 μm. Lower panel represents co-localized images from the time-series showing tracked single cell movement within tumors (green, 0 seconds; red, 100 seconds; blue, 300 seconds). White arrows indicate direction of cell movement. (**B**) Images represent Dendra2 labeled SCC FAK-wild-type expressing cells imaged using optical window chambers at 0, 6, and 24 hours post photo-switching (green, unswitched; red, switched). Scale bar 100 μm. Graph represents quantification of the total area covered by red fluorescence at sequential time-point following photo-switching.

Non-invasive, repeated imaging of single cells expressing photoswitchable probes in real-time has recently been used to examine breast cancer motility and intravasation [23]. This approach has facilitated in the assessment of therapeutic intervention on other tumor types, including the brain, and has also been utilized to monitor the cross-linking and quantity of surrounding ECM components at different stages of drug treatment within the same animal [24,25,72]. Cell tracking algorithms have commonly been used to provide quantitative analysis of cell migration *in vivo* by following fluorescent labeled cells (Figure 7A), however the statistical robustness of such methods are limited by the low number of events recorded. Simultaneous photoactivation of a population of Dendra2 expressing cells followed by temporal analysis of distribution patterns of labeled tumor cell populations provides a more robust quantitative assessment of *in vivo* tumor migration [71] (Figure 7B).

The application of specific fluorescent reporters of protein activity in conjunction with intravital microscopy enables specific pathway analysis *in vivo* with high spatial and temporal dynamics. Recent studies using a Smad2-GFP fusion reporter that translocates to the nucleus upon TGFb signaling was used to monitor TGFb mediated signaling in migrating Mtln3E tumor cells transplanted *in vivo* [73]. Time-lapse analysis reveals TGFb signaling activity and smad2-GFP nuclear localization in single moving cells, whereas, smad2 reporter remained cytoplasmic in cells invading in a collective manner or in non-motile cells [73]. This data implies that reversible TGFb signaling can regulate the mechanism of breast cancer cell invasion *in vivo* and further suggests transient TGFb signaling is an important determinant of tumor metastasis. Such transient events would be difficult to ascertain from fixed endpoint studies which can often lead to conflicting hypotheses in the absence of temporal information.

**Figure 8.**
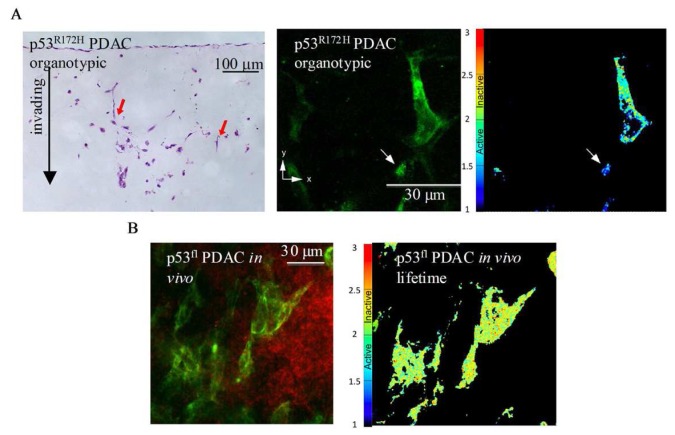
Protein activity monitored by multiphoton-based FLIM-FRET in 3D organotypic assays and *in vivo* live mouse model. (**A**) Left panel: H&E-stained sections of mutant p53^R172H^ -driven PDAC cell invasion within collagen I/fibroblast organotypic matrix culture. Middle panel: Representative fluorescence images of mutant p53^R172H^ cells expressing the Raichu-RhoA reporter (green) within the matrix during invasion (∼150 μm). Right panel: Corresponding life-time map of RhoA activity in mutant p53^R172H^ cells in the presence of a chemotactic gradient. Red arrows indicate representative cells examined at depth by multi-photon based FLIM-FRET. Low basal RhoA activity is represented in the life-time color maps with red/yellow colors, while high RhoA activity is represented as blue on the color map. (**B**) Protein activity monitored by multiphoton-based FLIM-FRET *in vivo*. Left panel: p53^fl^ PDAC cells expressing the RhoA-Raichu reporter (green) within a tumor. Blood vasculature is highlighted by tail vein injection of Qtracker 655 quantum dots (red). Right panel: Corresponding life-time map of RhoA activity *in vivo*.

A recent study using multi-photon based FLIM-FRET demonstrated for the first time the capacity to apply FLIM to detect sub-cellular activation of protein species upon therapeutic intervention in live animals [74]. These studies demonstrated the presence of a distinct fraction of RhoA protein activity at the poles of invasive pancreactic ductal adenocarcinoma (PDAC) cells expressing mutant p53^R172H^ protein [74]. Figure 8A displays images from organotypic cultures showing distinct regions of RhoA activity at the front of invading cells (Figure 8A middle and right panels). The preliminary finding that RhoA was spatially regulated during invasion was achieved using complimentary organotypic 3D matrices involving co-culture of pancreatic tumor cells on top of a fibrillar collagen I gel and embedded human fibroblasts (Figure 8A, left hand panel, H&E). This allowed the 3D manipulation and fluorescent lifetime imaging of RhoA activity during invasion to be investigated in a complex *in vitro* setting prior to *in vivo* examination of this biological process (see Figure 8A, middle and right hand panel). Importantly, this pool of RhoA activity at the front and rear of cells correlates with invasive potential *in vivo* and is absent in p53^fl^ null PDAC cells *in vivo* (Figure 8B, [74]). Such detailed spatial analysis of protein activity within individual cells provided by in *vivo* FLIM enables the quantification of dynamic molecular events operating under precise subcellular control at a single cell level and thus intractable to standard biochemical techniques. The application of such technologies as drug response biomarkers or surrogate markers of drug activity provide precise quantification of biochemical signals and pharmacological response *in vivo*. The applications of these technologies display early promise as a new type of kinetic pharmacodynamic biomarker for preclinical drug evaluation studies. Analysis of kinetic biomarkers using a variety of distinct technologies including live cell imaging offers several advantages over static markers such as more sensitive quantification of flux through biochemical pathways and dynamic phenotypic response in integrated living systems [75]. Application of such methods to high quality preclinical models of disease, including genetically engineered humanized mouse models that utilize Cre-lox recombination technology to recapitulate the genetics and pathophysiology of human disease and incorporate fluorescent reporters, further enhance preclinical evaluation under appropriate biological context [76-79].

## 6. Biosensors

The discovery and application of natural fluorescent proteins, such as green fluorescent protein (GFP), as functional biosensors of protein activity and cellular contrast agents has revolutionized the study of protein function, location and cell activity in intact cells and living organisms [80,81]. The development of engineered cell lines stably incorporating fluorescent protein reporter molecules has contributed to the increased throughput and application of both fixed endpoint and live cell high content analysis screens in drug discovery [82-84].

**Figure 9.**
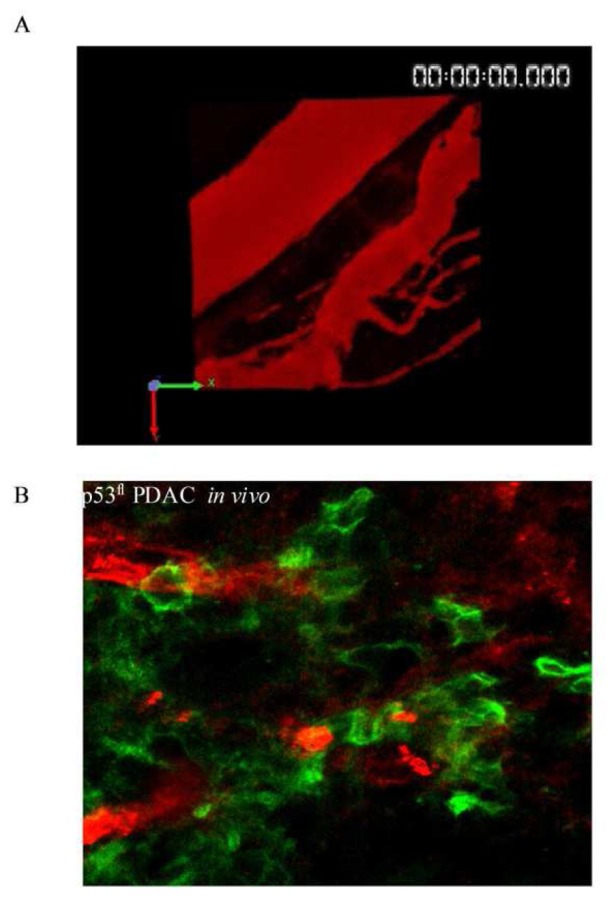
*In vivo* imaging with Quantum dots. (**A**) 3D rendered image of blood vasculature *in vivo* labeled by tail vein injection of mouse model with Qtracker 655 quantum dots; (**B**) Contextual imaging of cells *in vivo*. p53^fl^ pancreatic ductal adenocarcinoma cells expressing plasma membrane bound GFP (green) within an *in vivo* tumor in live mouse. Blood vasculature is highlighted in red by tail vein injection of Qtracker 655 quantum dots.

Recent developments in the optimization of optical probe design specifically tailored towards live cell imaging applications are advancing both the variety of applications and quality of experimental investigation. Such advances include genetic mutagenesis of fluorescent proteins to give rise to distinct spectral properties facilitating multiplexing or specialized spectroscopic techniques such as photoactivation or photoswitching [21,28,85]. The discovery and development of more stable monomeric red and far-red fluorescent proteins minimizes perturbation of protein structure and function in living systems while increasing the penetration of fluorescent signals through deep tissue [85-87]. Chemical optimization of fluorescent dyes based on organic small molecules has produced fluorescent probe conjugates with more efficient spectral properties for live cell imaging applications [88-90]. Further chemical design of peptide scaffolds that act as substrates for specific enzymes and which incorporate fluorescent reporters of enzyme activity have further expanded the application of image-based biosensors in live cell and live *in vivo* models [91-93]. Advances in nanotechnology have created light emitting nanoparticles, such as quantum dots that possess superior spectral properties and greater flexibility as functional reporters [94]. Quantum dots are small nanocrystals which exhibit high extinction coefficients and quantum yields that make them brighter and more photostable than fluorescent proteins or typical fluorescent dyes. The broad excitation and tunable emission properties of quantum dots enables the design of nanoparticles with broad excitation but narrow emission properties within the near infrared fluorescent (NIRF) window of optimal tissue penetration. High photostability, superior brightness, flexible NIRF emission wavelengths and thus increased sensitivity exhibited by quantum dots are ideal properties for deep tissue imaging *in vivo* and long term sequential imaging in live specimens [95]. Figure 9B demonstrates GFP labeled pancreatic ductal adenocarcinoma (PDAC) cells *in vivo* (green) with bloodvessels labeled by intravenous injection of quantum dots in red, thus, providing contextual information and orientation to the *in vivo* location of tumor cells (Figure 9).

The advances in fluorescent probe design described above provide a number of advantages for long term study in live-cell *in vitro, ex vivo or in vivo* models, such as, enhanced tissue penetration, reduced spectral overlap with tissue autofluorescence, reduced risks of phototoxicity, photobleaching and perturbation upon protein function. The commercial availability of expanding portfolios of optical imaging probes (Table 1) has contributed to a dramatic increase in the number of live cell imaging investigations that can be routinely performed. Such advances in probe design have been complemented by advances in multiphoton confocal microscopy and automated kinetic imaging technology for visualizing fluorescent reporters in live systems. Thus, the mutual benefits of fluorescent probe design and platform technologies for detecting fluorescent reporters are synergistic and have been instrumental in driving the most significant advances in the field of optical imaging over the past decade.

## 7. Conclusions

The latest advances in live cell imaging technologies and functional fluorescent reporters have stimulated the development of a number of innovative live-cell imaging techniques providing detailed temporal profiling of cell behavior and dynamic molecular events in complex *in vitro* and *in vivo* model systems. High resolution *in vitro* and intravital *in vivo* live imaging applications are heralding a new era of “high-definition” drug response profiling. Profiling drug candidates across dynamic *in vitro* and *in vivo* live imaging assays can provide unique insights into therapeutic mode-of-action and robust quantification of transient responses. Fluorescent based biosensors of cell phenotype and target activity *in vivo* can act as early surrogate proof-of-mechanism or proof-of-principal kinetic biomarkers, thus facilitating precise pharmacodynamic and efficacy studies directly within diseased tissue in live animals. The precise temporal and spatial information on cellular and tissue phenotypes provided by live imaging approaches provides additional biological context and refined temporal information for identifying genomic or proteomic profiles of pathophysiology and or drug response. Live cell imaging applications in drug discovery are largely target-agnostic and encompass large areas of biological space and thus ideal for opportunistic drug reprofiling or drug combination studies. Live cell imaging is also an ideal companion technology for leveraging additional value from genome-wide siRNA screens and emerging stem cell differentiation studies. A key future prospect of live imaging in drug discovery applications is uncovering precise and novel phenotypic information from complex *in vitro*, *ex-vivo* and *in vivo* systems. Thus, live-cell imaging may provide new insight into old drugs and old model systems. A critical issue for current drug discovery strategies is determining whether image-based endpoints provide greater predictive power of clinical response outcomes over standard preclinical measurements. Retrospective studies performed on approved drugs and clinical trial failures may help identify which image-based endpoints, biomarkers and experimental models are predictive of clinical outcome. It is likely that a multiparametric approach encompassing several image-based endpoints and a suite of models systems will deliver the optimal predictions of drug efficacy or at least facilitate clinical positioning, dosing strategies and thus more effective clinical trial designs.

## Figures and Tables

**Table 1.** Selected list of commercially available live cell imaging probes, applications and vendors.

**Probe**	**Application**	**Vendor**
Premo™ FUCCI Cell cycle sensor	Cell-cycle	Invitrogen
Premo™Autophagy Sensor (LC3B-RFP or GFP)	Autophagy	Invitrogen
Premo™Calcium Sensor	Ca2+ signaling	Invitrogen
CellLight™Histon2BGFP	Nuclear morphology	Invitrogen
CellLight™Actin/Tubulin GFP/RFP	Cytoskeletal dynamics	Invitrogen
CellLight™Talin GFP/RFP	Adhesion dynamics	Invitrogen
Human EGFR live cell fluorescent biosensor assay	EGFR signaling dynamics	Sigma
CompoZr cellular reporter cell lines beta-actin, tubulin alpha-1B chain, LamininB1, HMGA1	Cytoskeletal dynamics	Sigma
Lysotracker & LysoSensor	Lysosomes	Invitrogen
TMRE	Mitochondria function	Invitrogen
pHrodo™Indicators	Phagocytosis & Endocytosis	Invitrogen
NucView	Apoptosis (caspase activity)	Biotium
MitoView633	Mitochondrial function	Biotium
Sulforhodamine 101-annexin V apoptosis or TexasRedTM-annexinV	Apoptosis	Biotium
CellBrite Cytoplasmic membrane labeling kits (Neuro-DiO)	Cell Tracking	Biotium
DiO/DPA FRET pair	Membrane potential	Biotium
Cell Trace™ Violet	Cell proliferation	Invitrogen
MMPSense	MMP activity	PerkinElmer
ProSense	Cathespin B, L, S, plasmin activity	PerkinElmer
OsteoSense	Bone turnover	PerkinElmer
AnnexinVivo	Apoptosis	PerkinElmer
Angiosense & Angiospark(pegylated-nanoparticles)	Vascular Labeling	PerkinElmer
IntegriSense	Angiogenesis/tumor cell metastasis	Perkin Elmer
ReninSense	Renin activity	Perkin Elmer
Neutrophil Elastase	Neutrophil Elastase activity	Perkin Elmer
CatK	Cathepsin K proteinase activity	Perkin Elmer
CatB	Cathepsin B protease activity	Perkin Elmer
Qtracker quantum dots	Cell/vascular labeling	Invitrogen
FM 4-64	Membrane label	Invitrogen
Acrivlavin	cell label/mask	Sigma
Carbocyanine dye DiI, DiO	Membrane label	Invitrogen
Fluorescent lectins	Bloodvessel endothelium	Invitrogen
Fluorescent dextran	Vascular labeling	Invitrogen
